# Megalin–deficiency causes high myopia, retinal pigment epithelium-macromelanosomes and abnormal development of the ciliary body in mice

**DOI:** 10.1007/s00441-014-1919-4

**Published:** 2014-07-01

**Authors:** Tina Storm, Steffen Heegaard, Erik I. Christensen, Rikke Nielsen

**Affiliations:** 1 Department of Biomedicine, Faculty of Health Sciences, Aarhus University, Wilhelm Meyers Allé 3, DK-8000 Aarhus, Denmark; 2 Eye Pathology Institute, Department of Neuroscience and Pharmacology, University of Copenhagen, Copenhagen, Denmark; 3 Department of Ophthalmology, Glostrup Hospital, University of Copenhagen, Copenhagen, Denmark

**Keywords:** Megalin, Myopia, Non-pigmented ciliary body epithelium, Retinal pigment epithelium, Macromelanosomes

## Abstract

In man, mutations of the megalin-encoding gene causes the rare Donnai-Barrow/Facio-Oculo-Acoustico-Renal Syndrome, which is partially characterized by high-grade myopia. Previous studies of renal megalin function have established that megalin is crucial for conservation of renal filtered nutrients including vitamin A; however, the role of megalin in ocular physiology and development is presently unknown. Therefore, we investigate ocular megalin expression and the ocular phenotype of megalin-deficient mice. Topographical and subcellular localization of megalin as well as the ocular phenotype of megalin-deficient mice were examined with immunological techniques using light, confocal and electron microscopy. We identified megalin in the retinal pigment epithelium (RPE) and non-pigmented ciliary body epithelium (NPCBE) in normal mouse eyes. Immunocytochemical investigations furthermore showed that megalin localizes to vesicular structures in the RPE and NPCBE cells. Histological investigations of ocular mouse tissue also identified a severe myopia phenotype as well as enlarged RPE melanosomes and abnormal ciliary body development in the megalin-deficient mice. In conclusion, the complex ocular phenotype observed in the megalin-deficient mice suggests that megalin-mediated developmental abnormalities may contribute to the high myopia phenotype observed in the Donnai-Barrow Syndrome patients and, thus, that megalin harbors important roles in ocular development and physiology. Finally, our data show that megalin-deficient mice may provide a valuable model for future studies of megalin in ocular physiology and pathology.

## Introduction

The human *LRP2* gene encodes megalin, a 600-kDa multi-ligand endocytic receptor. Mutation of the *LRP2* gene causes the severe Donnai-Barrow Syndrome (OMIM # 222448) (Kantarci et al. 2007), which is a rare disorder characterized by a set of diverse and inconsistent clinical manifestations (Pober et al. 2009). More specifically, these include hypertelorism, large anterior fontanelle, agenesis of the corpus callosum and congenital diaphragmatic hernia (Pober et al. 2009). Low-molecular-weight proteinuria and high myopia, however, have been consistently observed in these patients (Pober et al. 2009). Recently, we showed that the underlying molecular mechanism behind the selective low-molecular-weight proteinuria is a dysfunction of megalin in reabsorption of filtered proteins in the kidney (Storm et al. 2012). It is becoming increasingly more evident that dysfunction of megalin is a key factor in the development of high myopia due to additional observations of myopia in megalin-deficient zebrafish (Veth et al. 2011). So far, however, the molecular mechanisms underlying the megalin-mediated high myopia have not been investigated.

Megalin expression has previously been investigated in the developing mouse eye (Assemat et al. 2005) as well as the adult rat (Zheng et al. 1994) and human eye (Lundgren et al. 1997). In the adult mammalian eye, megalin has been identified in the ciliary body and retinal pigment epithelia but no information concerning the subcellular localization was provided. In the kidney, immunocytochemical investigations have previously identified megalin in the apical brush border as well as in vesicles of the endocytic and recycling apparatus of the proximal tubular epithelium cells (Christensen et al. 1998).

Megalin is one of the largest members of the low-density-lipoprotein receptor family and highly expressed in the proximal tubular epithelium of the kidney (Christensen et al. 2012). Through the years, numerous ligands have been identified for megalin (for a comprehensive list of ligands, see Christensen et al. 2012). Megalin-mediated tubular reabsorption of plasma proteins filtered by the glomerulus is essential for maintaining homeostasis of vitamins and nutrients (Christensen et al. 2012; Storm et al. 2012). Besides the established endocytic function of megalin in the proximal tubules, megalin has furthermore been suggested to be important for mediating endocytosis in other specialized epithelia throughout the body (Christensen et al. 2012; Marzolo and Farfan 2011). So far, the physiological role of megalin has not been established in extra-renal tissues, including the ocular epithelia, nor has the pathological role of megalin in development of high myopia been analyzed.

Based on previous observations of patients and animals with no megalin, it is clear that megalin is important for normal ocular function. To further elucidate megalin’s role in ocular physiology, pathology and embryological development, we performed detailed investigations of ocular megalin expression and subcellular localization in normal mouse eyes in combination with histological investigations of the ocular phenotype of megalin-deficient mice.

## Materials and methods

Animal experiments and breeding were approved by the Danish Animal Experiments Inspectorate and performed in a certified animal facility according to their guidelines.

### Animals

Mice with genetic inactivation of megalin in early embryogenesis have previously been reported (Amsellem et al. 2010). These mice were generated through crossings of mice bearing floxed *Lrp2* alleles with mice transgenic for the Cre recombinase gene under control of the MORE (Tallquist and Soriano 2000) promoter. Breeding and genotyping of the MORE-Cre *Lrp2* strain were as previously described (Amsellem et al. 2010). All mice were maintained on a mixed C57BL/6–129/Svj background and sacrificed at the age of 13–17 weeks. Inactivation of the *Lrp2* gene in the retina and ciliary body was evaluated through immunohistochemistry on paraffin-embedded whole eyes sections. Age-matched Cre-negative littermates were used as controls and all experiments were performed with 3–6 mice in each group.

### Tissue preparation and microscopy

Mice were perfusion fixed via the heart with 2 % paraformaldehyde in 0.1 cacodylate buffer, pH 7.4 for immunohistochemistry. For light microscopy, eyes were enucleated and subsequently post-fixed intact in perfusion buffer for 1 h prior to paraffin embedding. Paraffin sections of 5 μm were cut on a Leica RM 2165 microtome (Leica, Ballerup, Denmark), heated and placed in xylene overnight prior to rehydration in graded alcohols. Rehydrated sections were heated in Tris-EGTA buffer for antigen retrieval in a microwave oven for approximately 10 min, cooled and incubated in 50 mM NH_4_Cl in 0.01 M PBS (pH 7.4) for 30 min. Sections were subsequently incubated with primary antibodies in 0.01 M PBS (pH 7.4) with 0.1 % skim milk powder (Merck, Darmstadt, Germany) and 0.3 % Triton X-100 (AppliChem, Darmstadt, Germany) followed by incubation with fluorophore-conjugated secondary antibody and mounted with cover slips using fluoroshield™ with propidium iodide (Sigma-Aldrich, Brondby, Denmark) for visualization of the eye tissue. Histological analyses and image acquisition were performed using an inverted confocal microscope (Zeiss LSM 510 META, Jena, Germany) and processed using Axiovision 4.8 and Adobe Photoshop 8.0 software.

For general histological inspection, sections were counterstained with Meier’s haematoxylin stain and examined in a Leica DMR microscope equipped with a Leica DFC320 camera. Images were transferred by a Leica TFC Twain 6.1.0 program and processed using Adobe Photoshop 8.0.

For electron microscopy, enucleated mouse eyes were bisected prior to post-fixation in perfusion buffer for 1 h. Eyes were further micro-dissected into blocks of tissue encompassing ciliary body- or retinal pigment epithelium, embedded in 12 % gelatin, infiltrated over night with 2.3 M sucrose in PBS buffer (pH 7.4) and ultimately frozen in liquid nitrogen.

Cryosections of approximately 60 nm were obtained with a Leica EM FC6 Cryoultramicrotome (Leica). For immunolabeling, cryosections were incubated overnight, at 4 °C, with primary antibody in buffer containing 2 % mouse serum followed by incubation, at 4 °C, for 2 h, with 10-nm gold-conjugated secondary antibody (BioCell, Cardiff, UK). Cryosections were then embedded in methylcellulose containing 0.4 % uranyl acetate and examined in a Philips CM 100 electron microscope (FEI, Hillsboro, OR, USA).

In general, control slides included sections in which primary antibodies were replaced by equimolar concentrations of IgG fractions or non-immunized serum.

### Estimation of melanosome volume

Ocular tissue was micro-dissected and prepared as described above for electron microscopy. From each animal, two tissue blocks were chosen by random sampling for further analyses. Using a Philips CM 100 electron microscope, 5–8 pictures of RPE cells from the same section were obtained at a magnification of ×5,700. Melanosome volume was estimated by point-sampling of linear intercepts using the mathematical principle of volume-weighted mean volume as previously described (Sorensen 1991). In short, assuming isotropy of the melanosomes their mean volume was estimated using the formula:

$$ {\overline{v}}_{\mathrm{V}}=\pi /3\times \varSigma {l_0}^3/ P\left(\mathrm{mel}\right)\times \left(\left({L}_{\mathrm{n}}\times 1000\right)/\left(3.00\times \mathrm{magnification}\right)\right) $$

Σ*l*_*0*_^*3*^: Sum of cubed intercept lengths; *P*(mel): Total number of melanosomes intercepted; *L*_n_: Length of ruler.

All graphs were constructed using software (GraphPad Prism 6.0 for Windows, San Diego, CA, USA).

### Ocular axial length measurements

Ocular tissue was prepared as described above for light microscopy. Paraffin embedded whole eyes where sectioned until appearance of *n*. opticus and pupil. Subsequently, paraffin sections of 2 μm were cut on a Leica RM 2165 microtome, heated and placed in xylene overnight prior to rehydration in graded alcohols. Sections were then prepared for general histological inspection as described above and examined in an Olympus BX-40 microscope equipped with a Nikon DS-Fi1 camera using a ×1.25 objective. Images were transferred by NIS Elements F 4.00 software and processed using Adobe photoshop 8.0 software. Axial lengths were determined on two separate sections from each animal, measured in mm based on a 2,000-μm scalebar and depicted in a graph.

### Antibodies

Sheep anti rat megalin (Moestrup et al. 1993), rabbit anti rat AQP4 (AQP-004) (Alomone Labs, Jerusalem, Israel), rabbit anti rat AQP1 (AB 2219) (Merck Millipore, Billerica, MA, USA), rabbit anti rat NaK Atpase (α_2_ isoform) (AB 9094) (Merck Millipore) and goat anti human NKCC1 (SC-21545) (Santa Cruz Biotechnology, Dallas, TX, USA).

#### Secondary

Alexa Fluor^®^-488 antibodies: donkey anti-rabbit, rabbit anti-goat and donkey anti-sheep (Invitrogen, Taastrup, Denmark). Colloidal gold-coupled antibody: donkey anti-sheep, 10-nm particles (BioCell).

## Results

### Megalin is expressed in the RPE and NPCBE cells of the adult mouse eye

**Fig. 1:**
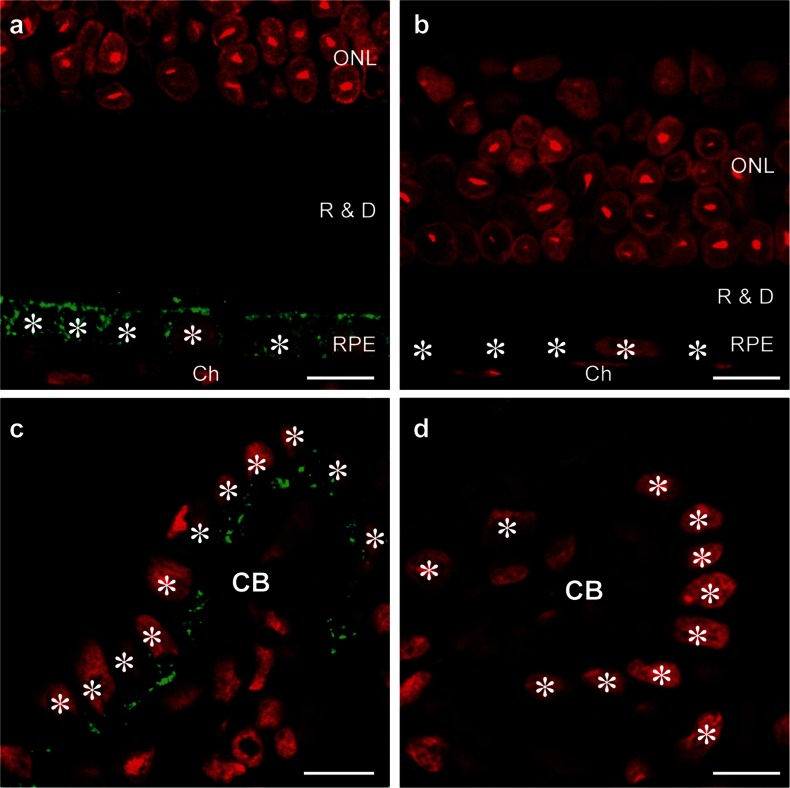
Immunohistochemical localization of megalin in mouse (normal (WT) (**a**, **c**)/megalin-deficient (KO) (**b**, **d**)). Megalin is shown in *green* and cell nuclei in *red*. Non-pigmented ciliary body and retinal pigmented epithelial cells are indicated with *white stars*. Megalin exhibits vesicular staining in the retinal pigment epithelium cells (**a**) and predominantly apical vesicular staining in the non-pigmented epithelium cells of the ciliary body (**c**). *ONL* outer nuclear layer, *R & D* rods and cones layer, *RPE* retinal pigment epithelium, *Ch* choroid, *CB* ciliary body. *Scale bars* 10 µm

**Fig. 2:**
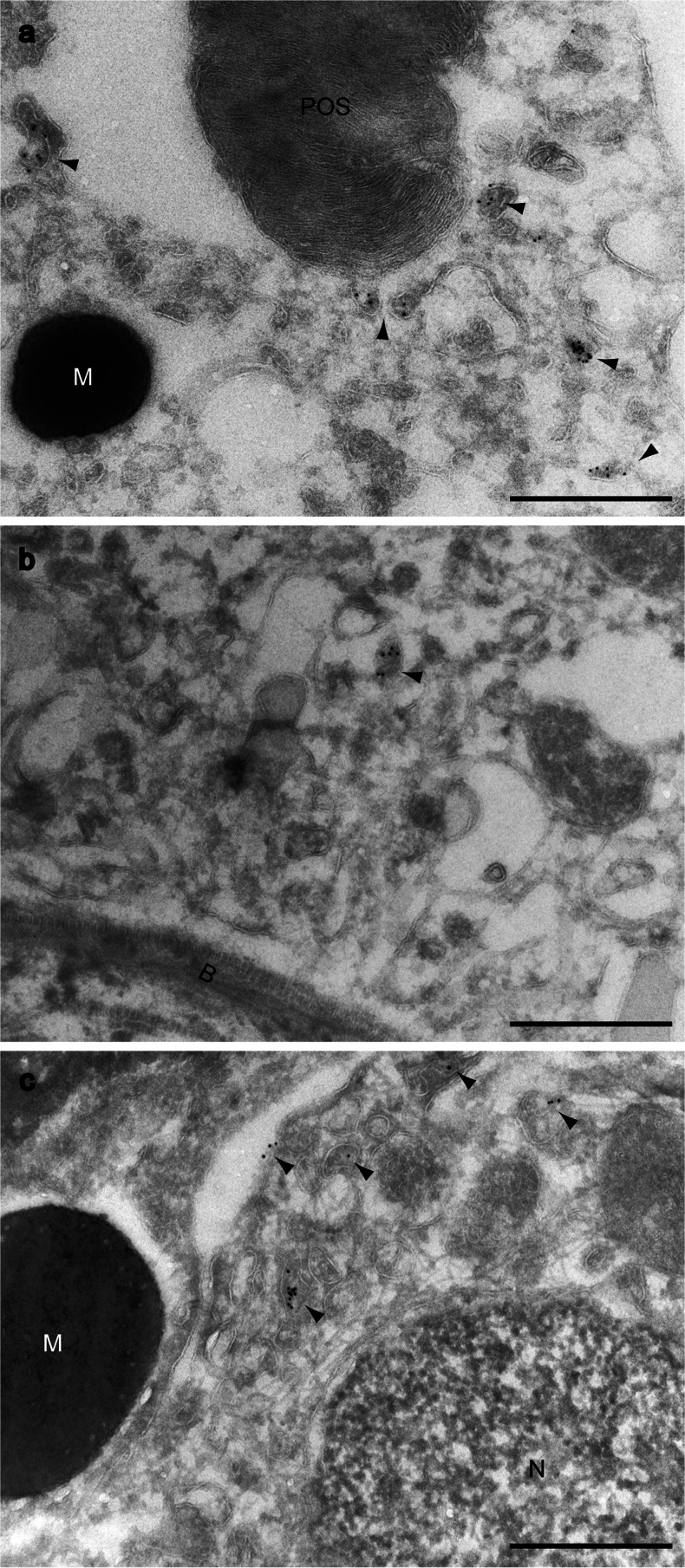
Immunocytochemical detection of megalin in ultrathin cryosections from mouse RPE and NPCBE cells. Immuno-gold labeling (*arrow heads*) shows that megalin localizes to vesicles of the endocytic apparatus in the apical (**a**) as well as the basolateral (**b**) regions of RPE cells. Megalin is furthermore detected in the apical membrane as well as the apical endocytic apparatus of NPCBE cells (**c**). *POS* photoreceptor outer segment, *M* melanosome, *B* basement membrane, *N* nucleus. *Scale bar* 0.5 µm

Immunohistochemical detection of megalin in normal mouse eyes (Fig. 1a, c) showed intense cytoplasmic labeling of the RPE and NPCBE cells. In RPE cells, a vesicular staining was observed throughout the cells (Fig. 1a). In NPCBE cells the staining appears to be concentrated to the apical part of the cells (Fig. 1c). Sections from megalin-deficient mouse eyes served as control for the specificity of the megalin antibody (Fig. 1b, d). Subsequently, the cellular localization of megalin was further investigated by immunocytochemistry on ultrathin cryosections of mouse RPE and NPCBE (Fig. 2a–c). Here, megalin was identified in vesicles in the apical as well as the basolateral regions of RPE cells (Fig. 2a, b). In contrast, megalin was only detected in apical vesicles of NPCBE cells (Fig. 2c).

### Mice deficient in megalin present with high myopia as well as RPEmacromelanosomes and abnormal ciliary body development

Efficiency of ocular inactivation of megalin in MORE-Cre *Lrp2* mice, where deletion occurs in the epiblast (Amsellem et al. 2010), was evaluated by immunohistochemistry on ocular tissue sections. Consistent with a previously reported efficiency of megalin kidney inactivation of 60–100 % in the MORE-Cre *Lrp2* model (Amsellem et al. 2010) no immunoreactivity for megalin could be detected in the majority of RPE and NPCBE cells (Fig. 1b, d).

**Fig. 3:**
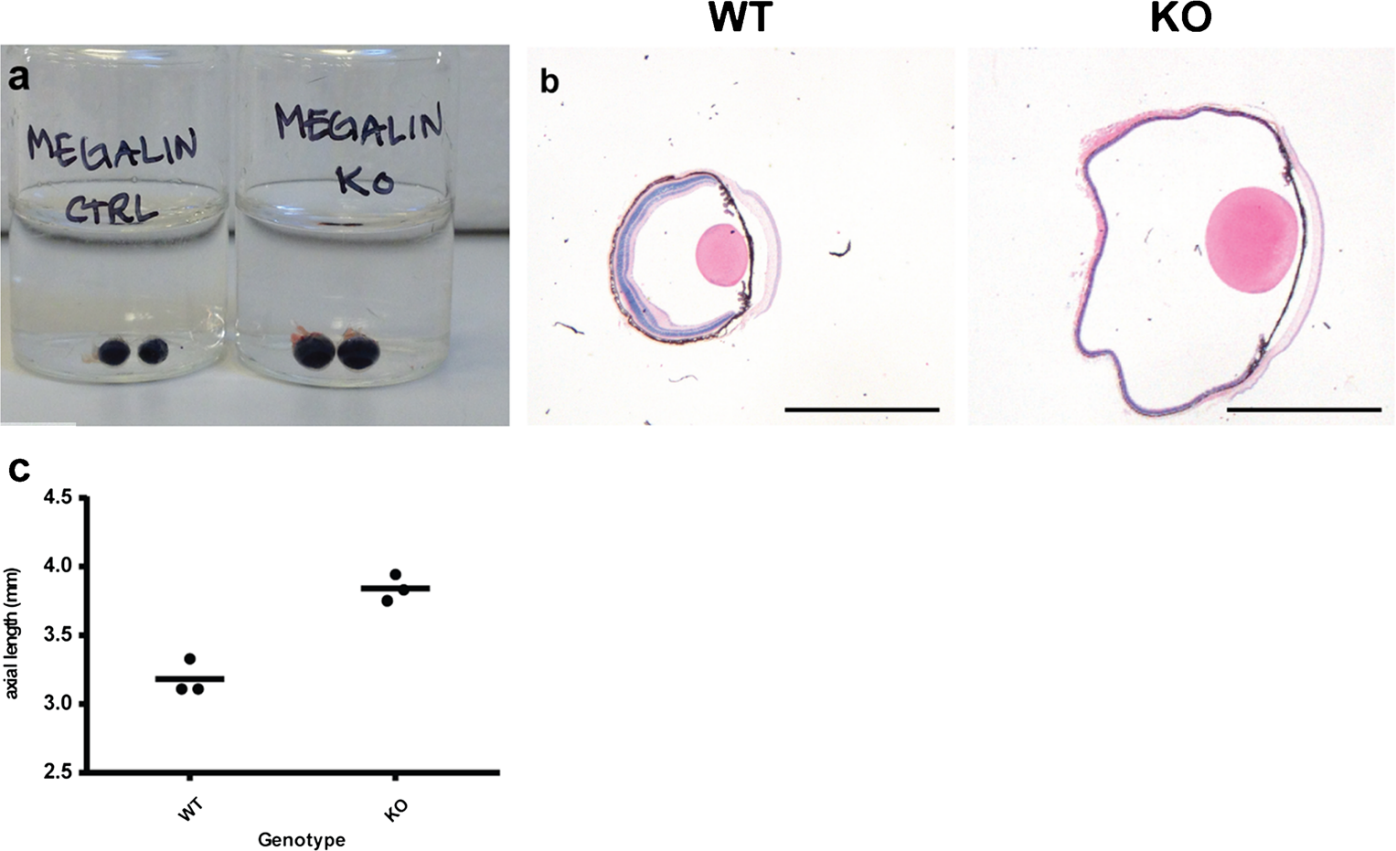
Difference in overall eye size in normal (WT) and megalin-deficient mice (KO). **a** Enucleated eyes from normal and megalin-deficient eyes. A large overall size difference can be observed between the normal eyes (*CTRL*) and the megalin-deficient (*KO*) eyes. **b** Histological cross-sections of normal (WT, *left*) and megalin-deficient (KO, *right*) mouse eyes. *Scale bars* 2000 µm. **c** Graph showing the axial length of eyes from normal (WT) and megalin-deficient (KO) mice. *Bars* represent median values. A large overall size difference can be observed between the normal and the megalin-deficient eyes. The axial length of the megalin-deficient eye is dramatically increased corresponding to a severe myopic refractive error in the megalin-deficient mouse eye

**Fig. 4:**
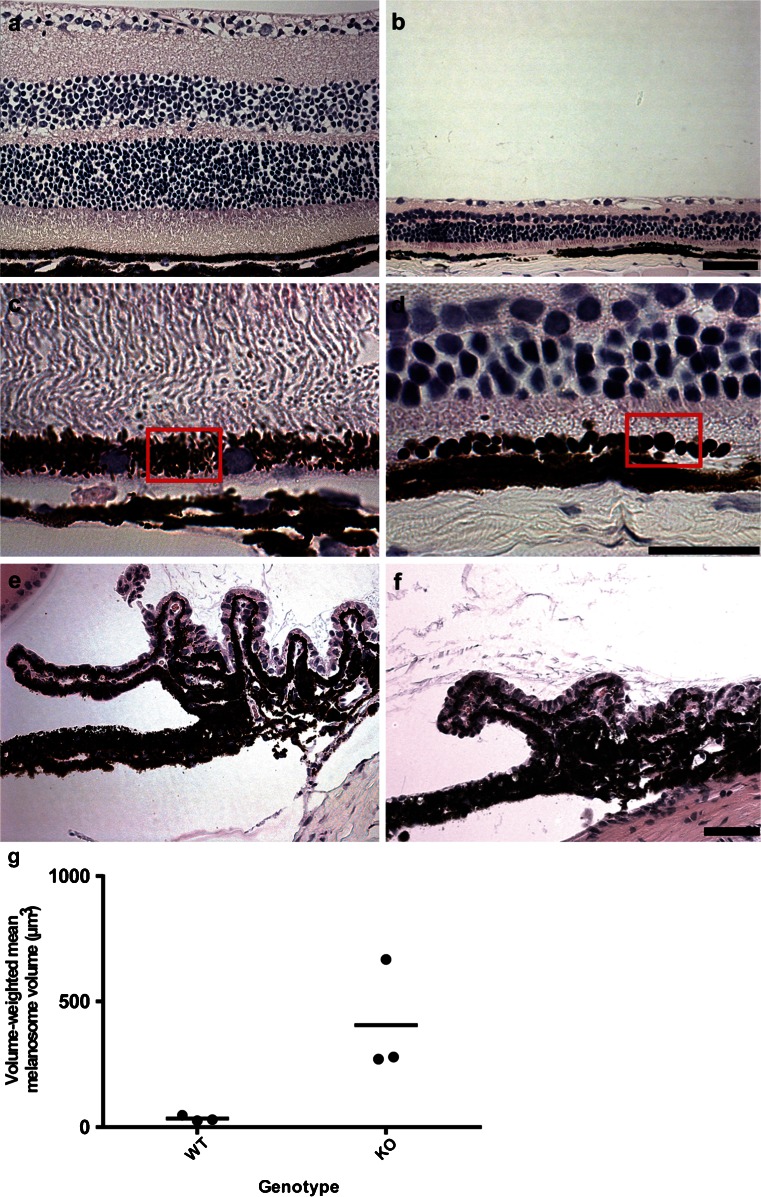
Histological cross-section of normal (WT, *left panel*) and megalin-deficient (KO, *right panel*) mice eyes. Severe thinning of all retinal layers (Hematoxylin-eosin staining, **a**, **b**, *scale bar* 50 µm), enlarged retinal pigment epithelium melanosomes (highlighted by *red squares*) (Hematoxylin-eosin staining, **c**, **d**, *scale bar* 20 µm) as well as a reduced number of ciliary processes (Hematoxylin-eosin staining, **e**, **f**, *scale bar* 100 µm), were observed in the megalin-deficient mouse eyes. **g** Graph showing the volume-weighted mean melanosome volume in RPE cells from normal (WT) and megalin-deficient (KO) mice. *Bars* represent median values

Overall, the megalin-deficient mice presented with a distinct and complex ocular phenotype. Enucleated eyes from the megalin-deficient mice showed an overt increase in overall eye size with a 1.2-fold axial length increase when compared to normal mouse eyes (Fig. 3a–c). Consistent with a severe myopic phenotype, we observed severe thinning of all retina layers in the megalin-deficient mice (Fig. 4a, b). Examination of whole-eye histological cross-sections also revealed enlarged melanosomes of the RPE cells in megalin-deficient mice (Fig. 4c, d). Stereological analyses of RPE cells by transmission electron microscopy revealed a 12-fold increase in mean volume of the melanosomes in megalin-deficient mice (Fig. 4g). However, no apparent difference in choroidal, iris epithelium or ciliary body pigment epithelium cell melanosomes was observed in the megalin-deficient mice.

**Fig. 5:**
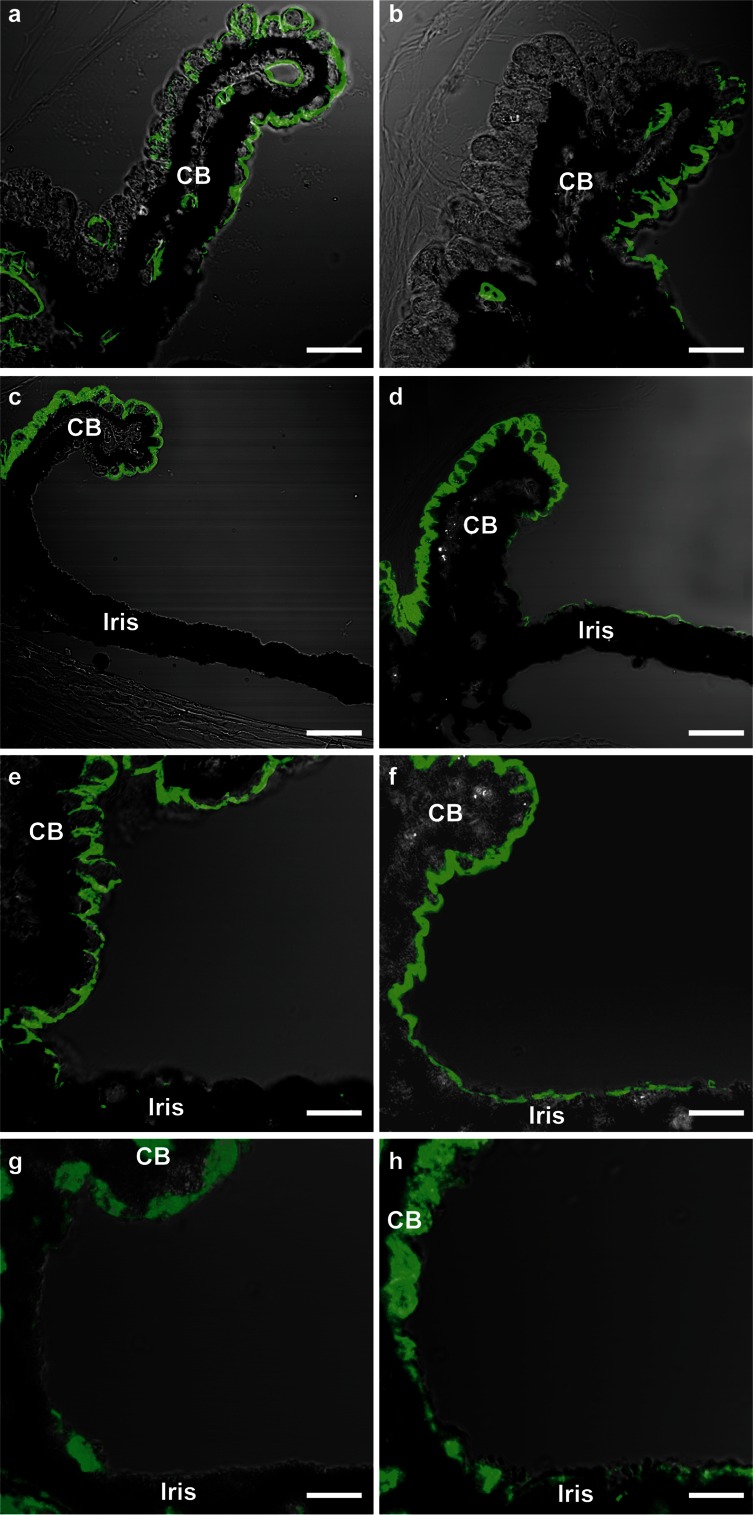
Immunohistochemical localization of selected water and salt transporters in ciliary body and iris epithelia. AQP1 (**a**, **b**), AQP4 (**c**, **d**), NaK ATPase (**e**, **f**), and NKCC1 (**g**, **h**). All are shown in *green*. An abnormal localization of AQP1, AQP4, NaK ATPase, and NKCC1 was observed in the megalin-deficient mice (KO) (**b**, **d**, **f**, **h**). CB ciliary body. *Scale bars* (**a**, **b**, **e**–**h**) 10 µm, (**c**, **d**) 20 µm

**Fig. 6:**
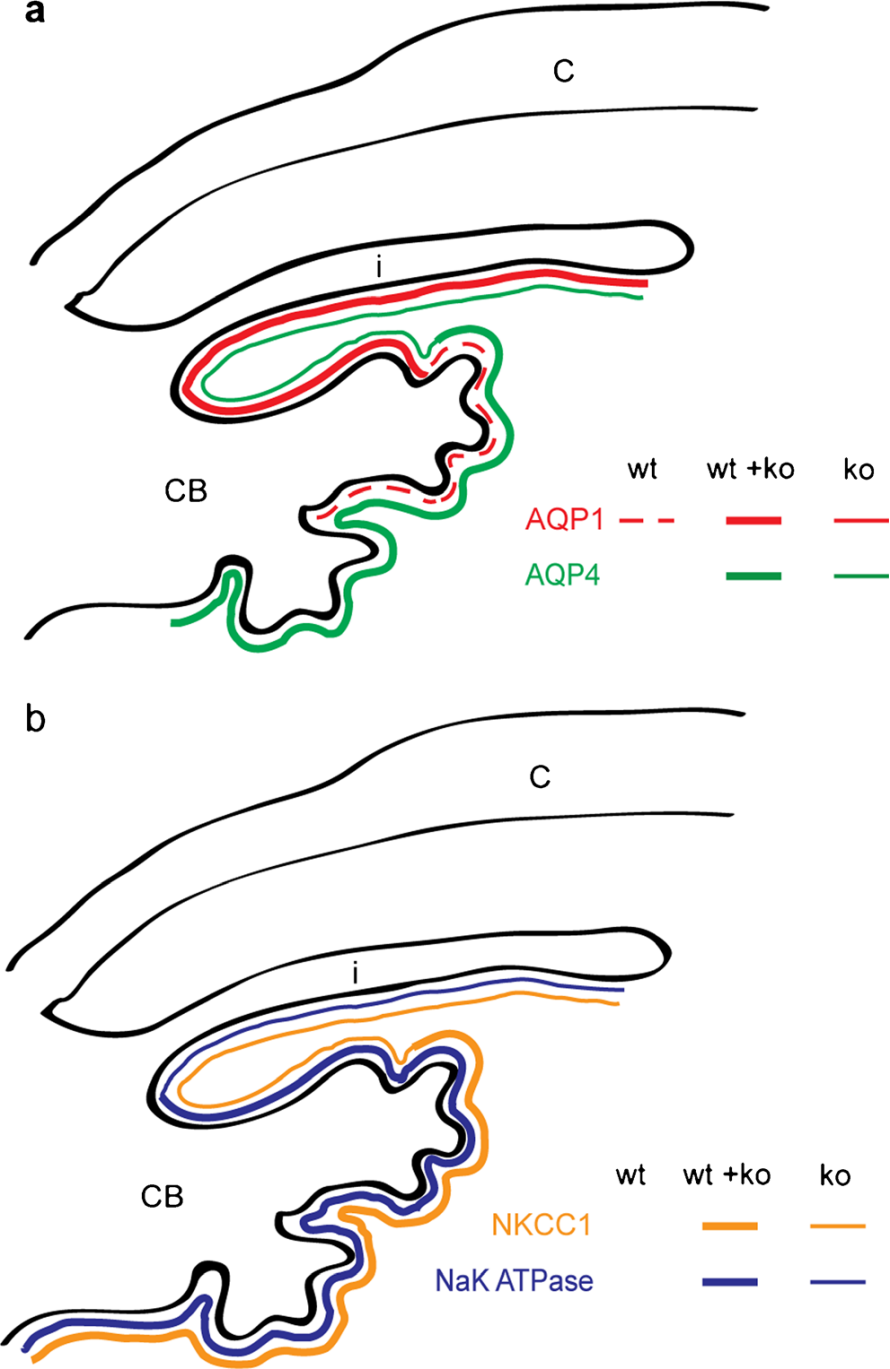
Illustration of the expression patterns for AQP1, AQP4 (**a**) and NaK pump, NKCC1 (**b**) in iris and NPCBE epithelia in normal (WT) and megalin-deficient (KO) mouse eyes. The extent of AQP1 expression is clearly reduced in the megalin-deficient mouse eyes. More specifically, the expression in the megalin-deficient mice is limited to the posterior iris epithelium and the NPCBE epithelium just adjacent to the iridocorneal angel (**a**). In contrast, the expression of AQP4, NaK pump, and NKCC1 are extended from an exclusive NPCBE expression, to also include the iridocorneal angle as well as the peripheral posterior iris epithelium (a b). C cornea, *i* iris, *CB* ciliary body

Interestingly, we also observed abnormal ciliary body development in the megalin-deficient mice. More specifically, the overall morphology of the ciliary body showed a reduced number of ciliary processes in the megalin-deficient mice (Fig. 4e and f) and detailed immunohistochemical investigations of the ciliary body revealed an atypical distribution of aquaporins (AQP) 1 and 4, the NaK ATPase (NaK pump) and the Na-K-Cl co-transporter (NKCC1) in megalin-deficient mice (Fig. 5). We observed that the distribution of AQP1 was limited to the iris epithelium and non-pigmented epithelium cells flanking the iridocorneal angle in megalin-deficient mice as opposed to the normal distribution including most non-pigmented epithelium cells of the ciliary body (Fig. 5a, b). In contrast, the expression of AQP4 (Fig. 5c, d), NaK pump (Fig. 5e, f) and NKCC1 (Fig. 5g, h) were extended beyond the normal NPCBE expression. In the megalin-deficient mice, expression of AQP4, the NaK pump and NKCC1 were also observed in the peripheral posterior iris epithelium (Fig. 5d, f, h). The expression patterns for AQP1, AQP4, NaK pump and NKCC1 in iris- and non-pigmented ciliary body epithelia in normal and megalin-deficient mouse eyes are summarized in Fig. 6a, b.

## Discussion

Histological analyses of eye tissue from normal mouse eyes revealed that megalin is expressed in the RPE and NPCBE and that it localizes to vesicular structures in these cells. We furthermore show that megalin-deficient mice display a distinct and complex ocular phenotype encompassing high myopia with severe retina thinning, abnormal ciliary body development and enlarged RPE melanosomes.

Our immunocytochemical investigations of RPE cells in mouse eyes revealed that megalin localizes to vesicular structures in the apical as well as to the basolateral region of RPE cells. Consistent with these findings, megalin has also been shown to localize to vesicular structures of the endocytic apparatus in other non-ocular epithelia (Kerjaschki and Farquhar 1983; Verroust et al. 2002; Nagai et al. 2003). In the proximal tubular epithelium of the kidney, numerous studies have, in addition, established that megalin is the dominating endocytic receptor (Christensen et al. 2012). RPE cells have also previously been shown to take up tracer molecules through coated pits in the apical as well as in the basolateral regions (Perlman et al. 1989). Together, this suggests that megalin may serve as an endocytic receptor in the RPE cells.

Detailed cytological investigations of NPCBE cells have previously identified an extensive pinocytosis of aqueous humor in the basolateral region of rats (Araki et al. 1993). However, we were only able to identify megalin in vesicular structures in the apical part of NPCBE cells, suggesting that megalin may mediate selective uptake of macromolecules from the blood to the NPCBE cells. To our knowledge, selective receptor-mediated endocytosis has not previously been described in the apical region of NPCBE cells. In the ciliary body, the capillaries are especially leaky and allow for the passage of macromolecules (Smith 1971; Uusitalo et al. 1973) but the composition of aqueous humor is significantly different from the blood. More specifically, the aqueous humor has lower protein content as well as a higher sodium and chloride content. Consistent with this, the NPCBE cells are connected through apical tight junctions and are believed to make up the so-called blood–aqueous humor barrier. Thus, our findings combined with the current knowledge on megalin function suggest that megalin may be a prime candidate for mediating a selective transport of vitamins and other nutrients across the blood–aqueous humor barrier delivering vital nutrients to the anterior structures of the eye.

Interestingly, megalin has also been proposed to be a key player in mediating sonic hedgehog (shh) and bone morphogenetic signaling (Bmp) in the developing brain (McCarthy et al. 2002; Spoelgen et al. 2005). Bmp signaling has furthermore recently been shown to be an essential pathway for ciliary body morphogenesis (Zhao et al. 2002; Zhou et al. 2013). In accordance with these findings, we identified abnormal ciliary body morphology in the megalin-deficient mice. In contrast to the 3–4 ciliary processes seen in normal mice (Napier and Kidson 2007; Napier and Kidson 2005), only 1–2 processes were observed in megalin-deficient mice. We also identified an atypical expression pattern of certain water channels and salt transporters in the NPCBE of megalin-deficient mice indicating that, in addition, the NPCBE cells could possibly be abnormally differentiated. Neither is observed in mice where the *Lrp2* gene is eliminated after the mouse is fully developed (unpublished data). Together, this therefore indicates that megalin is important for normal ciliary body development and, furthermore, that megalin may be a potential candidate for modulating Bmp signaling during ciliary body morphogenesis.

In contrast to the NPCBE cells, it is the basolateral region of the RPE cells that adjoins the vascular bed. As mentioned, we identified megalin in both the apical as well as in the basolateral regions of RPE cells suggesting that megalin may also be involved here in selective uptake of macromolecules from the blood.

We also identified megalin in the apical region of RPE cells. Based on the current knowledge of megalin function, one might further speculate that megalin could assist in the continuous exchange of visual cycle components between the photoreceptors and the RPE cells or possibly assist in RPE-retina signaling (Marzolo and Farfan 2011; Christensen et al. 2012). In the RPE, we observed a 12-fold increase in melanosome volume-weighted mean volume in megalin-deficient mice. The relationship between volume-weighted and number-weighted mean volume is: ν̅_V_ = ν̅_N_ × [1 + CV_N_^2^(ν)](Sorensen 1991). Thus, it is highly unlikely that the difference observed may be solely due to a different variation in melanosome volume in the knockout group (CV is the coefficient of variation of the number-weighted volume in each animal or intra-variation). Indeed, this would require a 33-fold higher intra-variation in the megalin-deficient animals compared to the normal mice. Interestingly, we did not observe macromelanosomes in the pigmented cells of the ciliary body nor the choroid. This could possibly reflect cell-specific functions and/or biogenesis of melanosomes as megalin is not expressed in either the pigmented cells of the ciliary body, iris epithelium, or the choroid. This therefore suggests that megalin may serve an important role in normal melanosome function and/or biogenesis in the RPE, although further studies are needed to determine the exact role(s).

To our knowledge, neither abnormal ciliary body development nor macromelanosomes have previously been associated with megalin dysfunction. Whether the severe myopic phenotype observed in fish, mice and patients with megalin dysfunction is due to developmental abnormalities remains to be established. Donnai-Barrow Syndrome patients (Pober et al. 2009) as well as megalin-deficient mice, are born with enlarged eyes indicating that megalin-mediated myopia may reflect developmental abnormalities. In contrast, however, megalin-deficient zebrafish gradually develop an adult onset myopia phenotype and furthermore present with a considerably less severe ocular phenotype (Veth et al. 2011). Our observations in the megalin-deficient mice thus appear to be more analogous to observations from the Donnai-Barrow Syndrome patients with respect to the severity as well as progression of the myopia (Patel et al. 2007; Kantarci et al. 2008; Pober et al. 2009; Schrauwen et al. 2013). The rarity of patients with megalin dysfunction combined with an absolute inaccessibility of ocular patient material to examine renders human studies of the underlying molecular mechanisms of megalin-mediated myopia impossible.

In conclusion, our data demonstrate that megalin serves important roles in ocular development and physiology. Also, the complex ocular phenotype observed in the megalin-deficient mice suggests that megalin-mediated developmental abnormalities may contribute to the high myopia phenotype observed in the Donnai-Barrow Syndrome patients. Finally, our data show that megalin-deficient mice may provide a valuable model for future studies of megalin function in ocular physiology, pathology and development.
